# Ohio State Dental Society

**Published:** 1922-12

**Authors:** 


### Ohio State Dental Society

THE attendance at the Convention of the Ohio State Dental Society held at the Gibson Hotel, December 5 to 7, 1922, was the largest ever registered at an Ohio State Convention. The local committee of Cincinnati dentists having this meeting in charge made elaborate plans for caring for the delegates and the result was a large meeting, good papers and clinics, much enthusiasm, fine dental displays, and big attendance. When Cincinnati does anything she does it well, was voiced by everyone. Many dentists from adjacent states, West Virginia, Kentucky, Indiana, and even Tennessee were attracted by the big dental show and helped to augment the large crowd in attendance. Many of the Dental Manufacturing Companies displaying at the Convention report satisfactory business besides its great educational value. The mezzanine floor as well as the ball room floor of the Gibson Hotel was utilized for display space so those who know the spaciousness of this hotel will appreciate the wonderful dental exposition which the dealers and manufacturers of Dental Supplies put on at this 57th Annual Meeting of the Ohio State Dental Society.

The following dental companies had displays at the meeting.

### LIST OF EXHIBITORS

**American Cabinet Co.,**
Two Rivers, Wis.

**Borine Mfg. Co.,**
551 W. 42nd St.,
New York City.

**Bristol Myers Co.,**
281 Green Ave.,
Brooklyn, N. Y.

**H. B. Byrd Mfg. Co.,**
300 Twelfth St.,
Washington, D. C.

**Colgate & Co.,**
199 Fulton St.,
New York City.

**L. D. Caulk Co.,**
Milford, Del.

**P. N. Condit,**
120 Boylston St.,
Boston, Mass.

**Cleveland Dental Mfg. Co.,**
Cleveland, Ohio.

**S. A. Crocker Co.,**
Cincinnati, Ohio.

**A. C. Clark Co.,**
1035 E. 76th St.,
Chicago, Ill.

**Columbus Dental Mfg. Co.,**
Wager & Jackson Sts.,
Columbus, Ohio.

**Denver Chemical Co.,**
20 Grand St.,
New York City.

**Dental Chemical Co.,**
Roslyn, Pa.

**Thomas J. Dee & Co.,**
5 So. Wabash Ave.,
Chicago, Ill.

**Detroit Dental Mfg. Co.,**
40-42 W. Milwaukee Ave.,
Detroit, Mich.

**The Kolynos Co.,**
New Haven, Conn.

**Dental Products Co.,**
5 S. Wabash Ave.,
Chicago, Ill.

**H. R. Lathrop Co.,**
116 Beekman St.,
New York City.

**Luthol Research Laboratories,**
Columbus, Ohio.

**Edwards X-ray Corporation,**
Indianapolis, Ind.

**Edral Co.,**
3306 Milton St.,
Cincinnati, Ohio.

**Electro Dental Mfg. Co.,**
33rd & Arch Sts.,
Philadelphia, Pa.

**Friedman Specialty Co.,**
22 E. Washington St.,
Chicago, Ill.

**Hugo Friedman Mfg. Co.,**
3660 Milwaukee Ave.,
Chicago, Ill.

**Ft. Wayne Dental Depot,**
Fort Wayne, Ind.

**Fellows Medical Mfg. Co.,**
26 Christopher St.,
New York City.

Robinson

**M. Gold,**
Cincinnati, Ohio.

**Horlick's Malted Milk Co.,**
Racine, Wis.

**Harvard Co.,**
Canton, Ohio.

**Hanan Engineering Co.,**
257 Washington St.,
Buffalo, N. Y.

**Heidbrink Co.,**
420 S. 6th St.,
Minneapolis, Minn.

**H. D. Justi & Son,**
1301 Arch St.,
Philadelphia, Pa.

**A. S. Koch & Sons,**

Lancaster, Pa.

**Dentists Supply Co.,**
220 W. 42nd St.,
New York City.

**Lavoris Chemical Co.,**
Minneapolis, Minn.

**Lambert Pharmacal Co.,**

21 Locust St.,

St. Louis, Mo.

**Dragon Products Co.,**
Columbus, Ohio.

**Dayton Dental Supply Co.,**
Dayton, Ohio.

**H. A. Metz Laboratories,**
122 Hudson St.,
New York City.

**Universal Dental Co.,**
Cor. 33rd & Columbia Ave.,
Philadelphia, Pa.

**Medical Protective Assn.,**
Fort Wayne, Ind.

**Mynol Chemical Co.,**
Real Estate Bldg.,
Philadelphia, Pa.

**Weisfeld Bros.,**
474 Broadway,
New York City.

**Wilmot Castle Co.,**
Rochester, N. Y.

**Pelton & Crane Co.,**
632 Harper Ave.,
Detroit, Mich.

**W. M. Ruthrauff,**
22nd & Arch Sts.,
Philadelphia, Pa.

**Ritter Dental Mfg. Co.,**
Rochester, N. Y.

**Liebel Flarsheim X-ray Co.,**
410 Home St.,
Cincinnati, Ohio.

**Rich-Alloy Co.,**
13223 Euclid Ave.,
Cleveland, Ohio.

**Senreco Corp.,**
304 Walnut St.,
Cincinnati, Ohio.

**Sodiphene Co.,**
928 Central St.,

Kansas City, Mo.

**Toledo Technical Appliance Co.,**

2226 Ashland Ave.,
Toledo, Ohio.

**Lochhead Laboratories,**
Chicago, Ill.

**Novocol Chemical Mfg. Co.,**
Brooklyn, N. Y.

**Lloyd F. Megaw,**

30 N. Michigan Blvd.,
Chicago, Ill.

**Victor X-ray Corp.,**
236 S. Robey St.,
Chicago, Ill.

**Weber Dental Mfg. Co.,**
Canton, Ohio.

**Chas. H. Phillips Chemical Co.,**
128 Pearl St.,
New York City.

**Poloris Co.,**
79 E. 130th St.,
New York City.

**H. B. Wiggins & Sons Co.,**
Bloomfield, N. J.

**Lee Smith & Son,**
Pittsburgh, Pa.

**Engeln Electric Co.,**
Cleveland, Ohio.

**Waite's Specialty Co.,**
Springville, N. Y.

**Ransom & Randolph Co.,**

Toledo, Ohio.

**S. S. White Dental Mfg. Co.,**
84 Market St.,
New York City.

**Sanitary Cup & Service Co.,**
317 N. Wells St.,
Chicago, Ill.

**Toledo Dental Supply Co.,**
Toledo, Ohio.

**Thwaites X-ray Co.,**
Grand Rapids, Mich.

**Dental Specialty Co.,**
1638 California St.,
Denver, Colo.

**Stratford Cookson Co.,**
42nd & Ludlow Sts.,
Philadelphia, Pa.

**John H. Wood Co.,**
126 Market St.,
Philadelphia, Pa.

